# Use of full blood count parameters and haematology cell ratios in screening for sepsis in South Africa

**DOI:** 10.4102/ajlm.v12i1.2104

**Published:** 2023-04-11

**Authors:** Jason van Rensburg, Saarah Davids, Carine Smuts, Glenda M. Davison

**Affiliations:** 1 Department of Biomedical Sciences, Faculty of Health and Wellness, Cape Peninsula University of Technology, Cape Town, South Africa; 2 South African Medical Research Council/Cape Peninsula University of Technology Cardiometabolic Health Research Unit and Biomedical Sciences, Faculty of Health and Wellness, Cape Peninsula University of Technology, Cape Town, South Africa; 3 Division of Haematology, Faculty of Health Sciences, University of Cape Town, Cape Town, South Africa

**Keywords:** sepsis, full blood count, neutrophil-to-lymphocyte ratio, monocyte to lymphocyte ratio, platelet-to-lymphocyte ratio

## Abstract

**Background:**

Sepsis is characterised by multi-organ failure due to an uncontrolled immune response to infection. Sepsis prevalence is increased in developing countries and requires prompt diagnosis and treatment. Reports, although controversial, suggest that full blood count parameters and cell ratios could assist in the early screening for sepsis.

**Objective:**

The study evaluated the use of haematological cell ratios in screening for sepsis in a South African population.

**Methods:**

The study retrospectively analysed the complete blood counts, blood cultures (BC) and biochemical test results of 125 adult patients who presented between January 2021 and July 2021 at a hospital in Cape Town. An ISO15189-accredited laboratory performed all of the tests. We compared and correlated the automated differential counts, neutrophil, monocyte and platelet-to-lymphocyte ratios with procalcitonin levels. A *p*-value of < 0.05 was considered significant.

**Results:**

Sixty-two sepsis patients (procalcitonin > 2 ng/L and positive BC) were identified and compared to 63 non-sepsis controls. All cell ratios were significantly elevated in sepsis patients (*p* < 0.001). However, the two groups had no significant difference in absolute monocyte counts (*p* = 0.377). In addition, no correlation was detected between any cell ratios and procalcitonin.

**Conclusion:**

In combination with complete blood count parameters, haematology cell ratios can be used for early sepsis detection. The full blood count is widely available, inexpensive, and routinely requested by emergency care clinicians. Although procalcitonin and BC remain the gold standard, the calculation of cell ratios could provide a simple screening tool for the early detection of sepsis.

**What this study adds:**

This study adds evidence to the proposal that calculating haematological cell ratios assists in the early screening of sepsis in a South African setting.

## Introduction

Sepsis is a life-threatening condition characterised by multiple organ system failures due to a dysregulated immune response manifesting secondary to a bacterial or fungal infection.^1^ Due to differing definitions of sepsis, global prevalence figures remain unclear; however, a minimum of 49 million sepsis cases are estimated to occur annually, with up to 11 million mortality.^2^ Sepsis prevalence is high in low- and middle-income countries due to high poverty levels and lack of healthcare resources. Immunocompromised individuals, the elderly and neonates are most at risk.^3^ Sepsis is a medical emergency that requires prompt treatment to achieve a favourable clinical outcome.

Clinical symptoms are often non-specific making diagnosis difficult, variable and unclear. The diagnosis is predominantly based on the sequential organ failure assessment score, which measures the functional status of the respiratory, haemostatic, hepatic, renal, cardiovascular and nervous systems.^4,5^ The sequential organ failure assessment score is not specific to sepsis; thus, biological markers such as blood cultures (BC), procalcitonin, and C-reactive protein have been adopted in clinical practice.^6^ These biomarkers, although important, have limitations.

Procalcitonin is a precursor molecule to the thyroid hormone calcitonin and is undetectable in the serum of healthy individuals. Procalcitonin levels correlate with sepsis severity, with serum concentrations between 0.5 ng/L and 2.0 ng/L indicative of bacterial infection and those > 2.0 ng/L indicative of sepsis.^7,8^ Although highly specific, procalcitonin assays may not be widely available, have a longer turnaround time than a complete blood count and require specialised instrumentation and costly reagents. Additionally, procalcitonin may be falsely elevated in conditions such as renal impairment, congestive heart disease, burn wounds, malignancies and acute pancreatitis, and all common sepsis complications.^9^

C-reactive protein is an acute-phase protein synthesised by the liver in response to inflammation, making it an early and sensitive marker of sepsis.^10^ C-reactive protein is elevated in infection, injury or chronic disease inflammations. Thus, it is not specific, limiting its use as a marker of sepsis.^11^

On the other hand, BC is a specialised investigation that can detect microorganism growth in a patient’s blood. It uses an enriched growth medium to support microbial growth and a colourimetric detection system to detect carbon dioxide produced by the growing blood pathogens.^12^ Any growth within the media indicates sepsis; thus, BCs are considered the gold standard of sepsis diagnosis. However, this test has limitations, including sample contamination due to non-sterile collection. Also, antibiotics use before sampling may cause false results. In addition, although clinicians get results within 24 h, slow-growing organisms can take 5 days or longer to grow, which is unsuitable for early diagnosis.^13^

Therefore, although these biomarkers and culture techniques are helpful, the need for a quick, efficient and cost-effective method to screen for early sepsis is still relevant. Recent studies have reported using full blood count parameters and cell ratios to screen for sepsis.^14,15^

It is generally accepted that an abnormal white cell count with an increase in immature granulocytes indicates sepsis and has been included as one of the four Systemic Inflammatory Response Syndrome criteria.^16^ Automated full blood and differential counts are essential in diagnosing sepsis and are often the first laboratory test to be requested. It has a fast turnaround time, is cost-effective and can potentially provide crucial information on peripheral blood cell populations if interpreted correctly. Bacterial sepsis is complex and results in the initiation of an acute inflammatory response with the release of cytokines, chemokines and alterations in the proportions and numbers of innate and adaptive immune cells. These changes can be detected by analysing the automated full blood count and differential results.^17^

Neutrophils, the most common peripheral leucocytes, form part of the innate immune response and are released by the bone marrow in response to several stimuli, including stress, smoking and inflammation.^18^ During sepsis or infection, they increase rapidly and can eliminate pathogens by phagocytosis and oxidative bursts. In severe cases, the neutrophil function can become dysregulated, leading to chronic inflammation.^18^ It is believed that after exposure to lipopolysaccharides, anti-apoptotic signals of the B-cell lymphoma 2 (BCL-2) family are upregulated, leading to neutrophils resisting apoptosis and increasing their lifespan.^18^

Although a disruption in neutrophil numbers can indicate infection, on their own they are not a reliable indicator of sepsis. In Africa, benign ethnic neutropenia, defined as a neutrophil count of < 1.5 × 10^9^/L in normal healthy individuals, is common, and researchers have proposed that the reference interval for neutrophils in African populations is lower than in other population groups.^19,20^ This African neutrophil reference interval is essential for benchmarking limits at which the white cell count and neutrophil count can screen sepsis in African populations.

On the other hand, lymphocytes make up 20% – 40% of circulating blood cells and form part of the adaptive immune system. During sepsis, this cell population, unlike neutrophils, undergoes apoptosis leading to severe lymphopenia. Therefore, calculating the neutrophil-to-lymphocyte ratio (NLR) can be a far more valuable and reliable sepsis biomarker than the neutrophil count alone.^21,22,23,24,25^

Monocytes, also measured by an automated cell counter, are innate immune cells that function as phagocytes and initiate the adaptive immune response by presenting bacterial antigens to T-cells and secreting proinflammatory cytokines. Monocyte counts are known to climb rapidly during the early stages of infection; however, much like neutrophils they can become defective and undergo rapid apoptosis.^26,27^ It has been suggested that the monocyte-to-lymphocyte ratio (MLR) could add valuable information to early sepsis screening.^28^ However, studies examining this parameter are scarce and conflicting.^29,30^

Platelets, the smallest circulating blood cells, lack a nucleus and are usually released by the bone marrow in response to bleeding and endothelial injury. Platelets participate in haemostasis and the inflammatory response by secreting cytokines, recognising pathogen-associated molecular patterns and activating innate immune cells.^31^ Thus, their involvement in both processes makes them critical in sepsis. Thrombocytopaenia, defined by a platelet count of < 150 × 10^9^/L, is common in sepsis and forms part of the sequential organ failure assessment score,^4^ which measures organ failure and forms part of the diagnostic criteria for sepsis. Furthermore, the prognostic value of a severely decreased platelet count (< 50 × 10^9^/L) in septic patients has been demonstrated,^32^ while a study of 5537 patients with sepsis reported that patients with an elevated platelet-to-lymphocyte ratio (PLR) at diagnosis (> 200) were associated with increased mortality.^33^

The calculation of the NLR, MLR and PLR are potential rapid screening tools to detect sepsis. Although studies have demonstrated the value of these ratios, reports have been variable and often unclear. Therefore, this study aimed to evaluate the utility of haematological cell ratios to detect Gram-negative bacterial sepsis in patients presenting to a hospital in Cape Town, South Africa, and to correlate the values with serum procalcitonin levels.

## Methods

### Ethical considerations

This study was retrospective and thus no additional testing was conducted on participants. Participant consent was waived and ethical clearance was granted by the Cape Peninsula University of Technology’s Ethics Committee (clearance number: CPUT/BMS-EC 2021/E32). Permission to use the laboratory’s patient records was obtained from the laboratory. All patient records were handled according to the Declaration of Helsinki and confidentiality was maintained by removing all patient identifiers. Data were stored on a password-protected computer which was only accessible to the primary investigator.

### Study design

This retrospective study analysed 125 adult patients (all > 18 years of age) with complete blood count parameters. The tests were conducted between January 2021 and July 2021 at an ISO15189-accredited laboratory in Cape Town, South Africa. Based on procalcitonin levels (> 2 ng/L) and BC results (positive with confirmed Gram-negative bacillary growth), participants were divided into two groups comprising those with confirmed sepsis (*n* = 62) and a control group (*n* = 63) which comprised hospitalised patients who had no clinical or laboratory evidence of sepsis. The haematological cell ratios and full blood count parameters were then compared and correlated using statistical tests.

### Full blood count and haematological cell ratios

Peripheral blood was collected into vacutainer tubes containing ethylenediaminetetraacetic acid. Automated full blood and white cell differential counts were performed by flow cytometric analysis on the Sysmex XN series automated analyser (Sysmex Corporation, Kobe, Hyogo, Japan). The NLR and MLR were calculated by dividing the relevant absolute cell line count by the absolute lymphocyte counts, whereas the PLR was calculated by dividing the total platelet count by the absolute lymphocyte count.

### Biochemical tests

Peripheral blood was collected into a serum separating tube and centrifuged at 4000 revolutions per minute for 5 to 10 min to remove the serum. Procalcitonin values were then generated using the chemiluminescent microparticle immunoassay principle and processed on the Abbott Alinity CI and Architect CI analysers (Abbott Laboratories, Chicago, Illinois, United States). C-reactive protein results were generated by immunoturbidimetry chemistry assay (Abbott Laboratories, Chicago, Illinois, United States). A procalcitonin cut-off value of > 2 ng/L was used to determine if a patient had sepsis.^8^

### Blood cultures

Blood culture samples were collected in duplicate for anaerobic and aerobic cultures. Cultures were incubated and monitored for growth for 7 days on the BioMérieux BactAlert 3D system (BioMérieux, Marcy-l’Etoile, France). Cultures flagged for growth by the analyser were examined microscopically and subcultured on appropriate media based on organism morphology and Gram stain results. Culture plates were examined after 18 h to 24 h of incubation, and isolates were speciated by mass spectrometry using a BioMérieux Vitek-MS’s (BioMérieux, Marcy-l’Etoile, France) principle of Matrix-Assisted Laser Desorption Ionization Time-of-Flight. In cases where inadequate microbial material was available for mass spectroscopy, biochemical analysis and speciation were performed using the BioMérieux Vitek 2 (BioMérieux, Marcy-l’Etoile, France). Only cultures with Gram-negative bacilli growth were considered for the sepsis group, as Gram-positive cocci are most likely to be pre-analytical contaminants.

### Data collection and statistical methods

All archived full blood counts, differential counts and blood chemistry data were collected from the laboratory’s information system. Consecutive patients, > 18 years of age, with a positive BC and a negative Gram stain, were included in the sepsis group, while the controls comprised hospitalised patients with no evidence of sepsis and negative BC. Results were collated on Microsoft Excel 2019 (Microsoft Corporation, Redmond, Washington, United States) and imported into jamovi version 2.3 (jamovi project, Sydney, Australia) for statistical analysis. Subjects were assigned unique identifiers, and only haematology, chemistry, culture results, age and gender data were collected to prevent bias and protect confidentiality. The normality of the data set was established by jamovi’s frequency statistics, with the range of −1 to 1 considered to be normally distributed. Skewed data were presented in quartiles, and significance was assessed using the Kruskal-Wallis’s test.^34^ Normally distributed data were presented as standard deviations, and significance was determined using the one-way analysis of variance test with *p* < 0.05 considered significant. Correlations between analytes were assessed by bivariate regression, and a *p*-value of < 0.05 was considered significant. Receiver operating characteristic curves were constructed to evaluate cell ratios as possible diagnostic screening markers for sepsis.

## Results

### General characteristics

The study comprised 125 patients’ data. Patients were over 18 years; 62 had sepsis, while 63 had no sepsis (control group). The 62 sepsis patients had Gram-negative bacteria (*Pseudomonas aeruginosa, Klebsiella pneumoniae, Escherichia coli* and *Serratia marcescens*) isolated from their BC and had a procalcitonin value > 2 ng/L. Of the 62 patients, 32 were male, and the group’s median age was 65 (range: 30–87). The results were compared to 63 controls over 18 years (19–77) with no evidence of sepsis (Table 1).

**TABLE 1:** Demographic data of sepsis and non-sepsis patients analysed between January 2021 and July 2021 in Cape Town, South Africa.

Variable	Sepsis (*n* = 62)		Non-sepsis (*n* = 63)		*p*
	Median	25th; 75th percentile)	Median	25th; 75th percentile)	
Age (years)†	62.9	13.5	49.4	18.6	< 0.001
Procalcitonin (ng/L)	15.2	6.84; 38.00	‡	‡	-
C-reactive protein (mg/L)†	211	108	‡	‡	-
Leukocyte count (× 10^9^/L)	13.4	6.96; 19.80	7.39	6.65; 8.58	< 0.001
Neutrophils Abs (× 10^9^/L)	11.90	5.68; 18.10	4.57	3.66; 5.00	< 0.001
Lymphocytes Abs (× 10^9^/L)†	0.86	0.74	2.22	0.61	< 0.001
Monocytes Abs (× 10^9^/L)	0.655	0.313; 1.020	0.560	0.450; 0.690	0.377
Platelets (× 10^9^/L)	172	87; 241	281	277; 314	< 0.001
Neutophil lymphocyte ratio	17.50	7.99; 27.60	2.03	1.60; 2.60	< 0.001
Monocyte-lymphocyte ratio	0.795	0.492; 1.400	0.260	0.200; 0.355	< 0.001
Platelet-lymphocyte ratio	129	111; 407	212	105; 157	< 0.001

Abs, absolute count.

† , Mean (standard deviation);

‡ , Data not available.

### Haematology

Patients with sepsis had a significantly elevated white cell and neutrophil count compared to the control group (*p* < 0.001). The absolute lymphocyte count was significantly reduced in the sepsis group (*p* < 0.001) and, similarly, the median platelet count was lower (*p* < 0.001). In contrast, although the median absolute monocyte counts varied, there was no statistical significance in monocyte counts between the sepsis and control groups (*p* = 0.377) (Table 1). All three cell ratios varied significantly between the two study groups, most notably the median NLR being 8.62 times higher in the sepsis group, whereas the MLR and PLR were 3.06 and 0.71 times higher. The differences in cell ratios were statistically significant, with *p* < 0.001 (Table 1).

### Neutrophil-to-lymphocyte ratio

**FIGURE 1:**
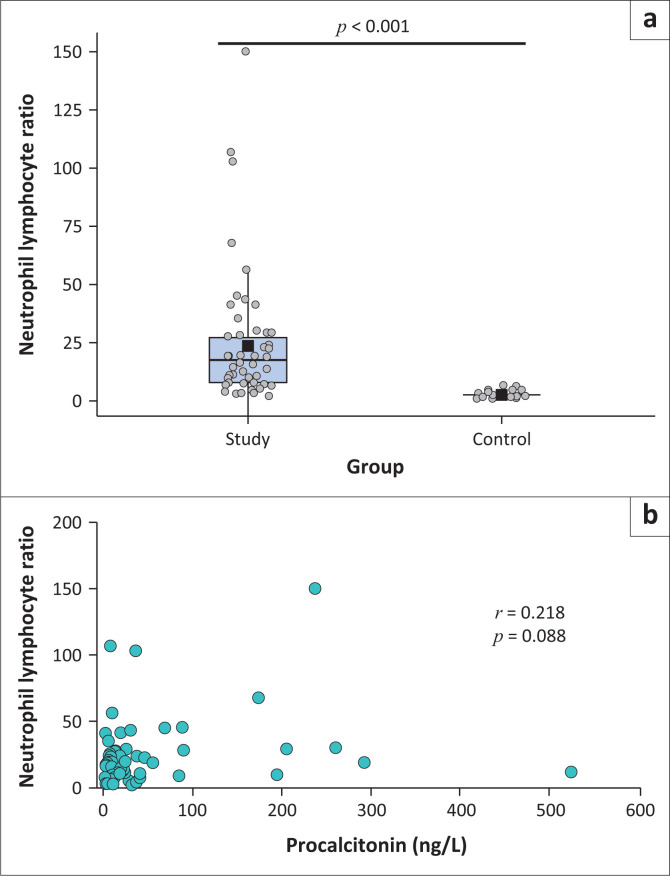
Neutrophil-lyphocyte ratio characteristics among 62 patients with sepsis compared to 63 non-sepsis controls presenting between January 2021 and July 2021 in Cape Town, South Africa. (a) The median and interquartile range of the neutrophil-lymphocyte ratio. (b) The correlation between the neutrophil-lymphocyte ratio and procalcitonin.

Although the NLR varied in those with sepsis, values were statistically significantly elevated (*p* < 0.001) compared to the control group (Figure 1a), which all fell within the reported NLR reference interval of 0.78 to 3.53 with a mean of 1.65.^21^ Despite the high levels of both NLR and procalcitonin, no correlation was detected between the two tests (*r* = 0.218; *p* = 0.088) (Figure 1b).

### Monocyte-to-lymphocyte ratio

**FIGURE 2:**
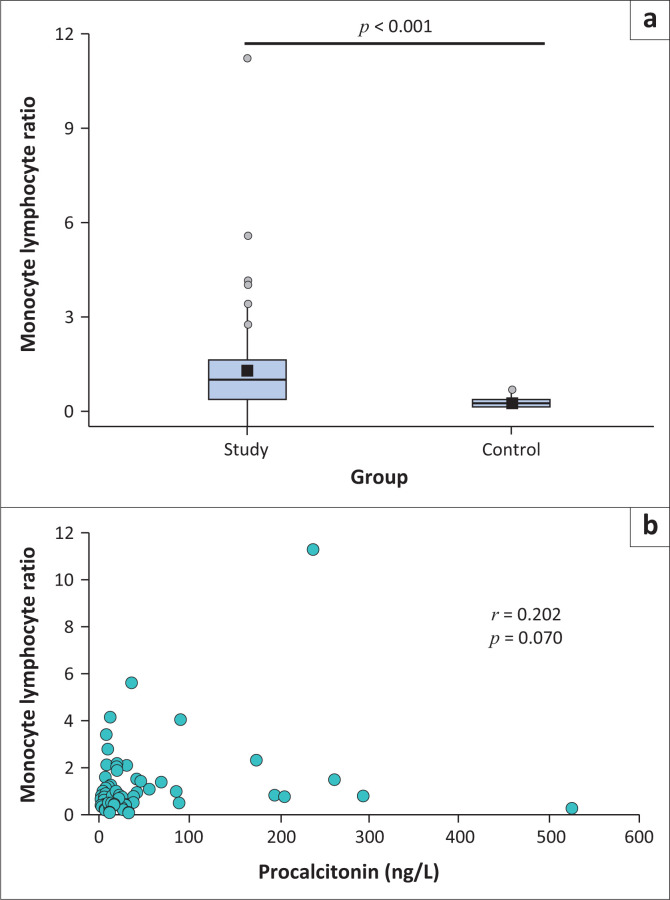
Monocyte-lymphocyte ratio characteristics among 62 patients with sepsis compared to 63 controls diagnosed between January 2021 and July 2021 in Cape Town, South Africa. (a) The median and interquartile range of the monocyte-lymphocyte ratio. (b) The correlation between the monocyte-lymphocyte ratio and procalcitonin.

Similarly, although the MLR showed a large variation, it was significantly elevated when compared to the non-septic control group (*p* < 0.001) (Figure 2a). No significant correlation could be detected between the MLR and procalcitonin levels (*r* = 0.202; *p* = 0.07) (Figure 2b).

### Platelet-to-lymphocyte ratio

**FIGURE 3:**
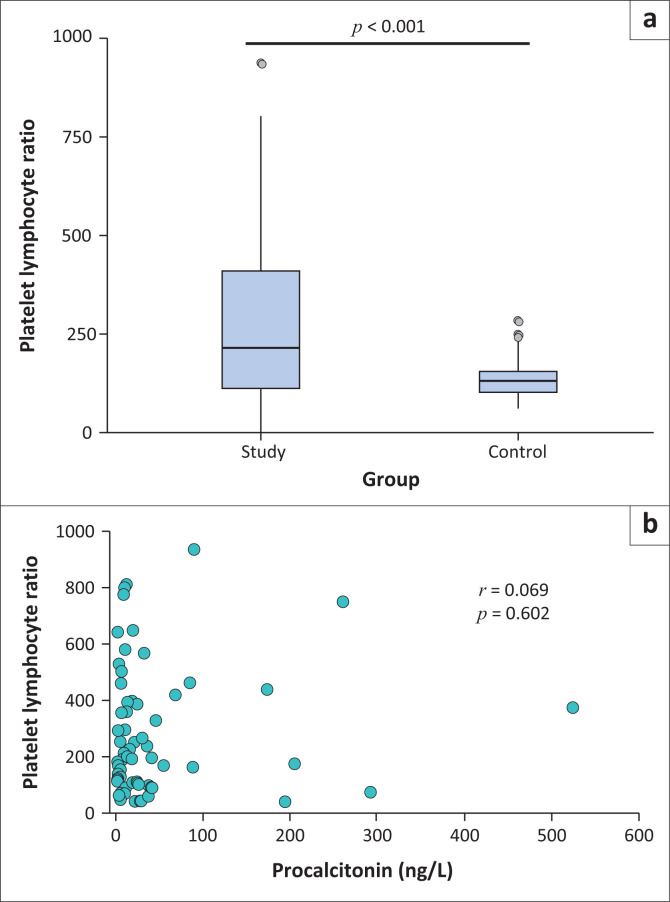
Platelet-lymphocyte ratio characteristics among 62 patients with sepsis compared to 63 controls presenting between January 2021 and July 2021. (a) The median and interquartile range of the platelet-lymphocyte ratio. (b) The correlation between the platelet lymphocyte ratio and procalcitonin.

The PLR in those with diagnosed sepsis was significantly higher than in the control group (*p* < 0.001) (Figure 3a); however, no significant correlation could be detected between the PLR and procalcitonin levels (*r* = 0.069; *p* = 0.602) (Figure 3b).

### Receiver operating characteristic curve analysis

**FIGURE 4:**
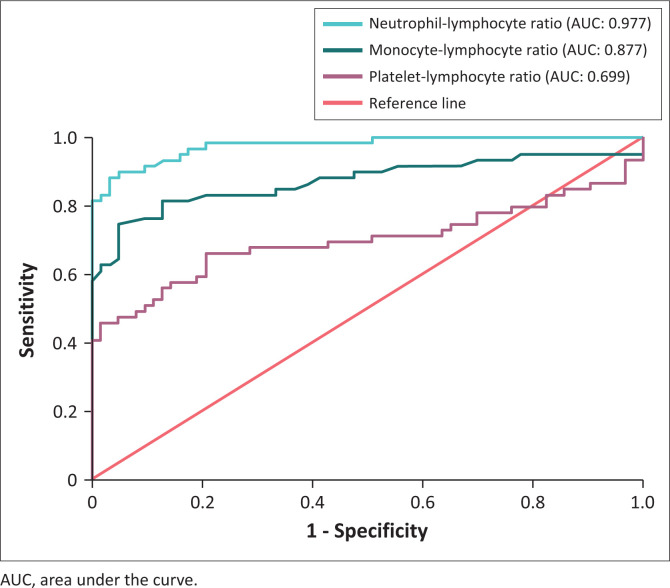
Receiver operating characteristic curves for the neutrophil-lymphocyte ratio, monocyte-lymphocyte ratio and the platelet-lymphocyte ratio of patients analysed between January and July 2021 in Cape Town, South Africa.

Among the three cell ratios assessed, the NLR was established as the best diagnostic screening marker (area under curve: 0.977; Youden index: 0.855), while the MLR was considered to be good (area under curve: 0.877; Youden index: 0.710). The PLR only performed moderately (area under curve: 0.699; Youden index: 0.455) (Figure 4).

## Discussion

In this retrospective study of 62 sepsis and 63 control patients, the NLR, MLR, PLR, white blood cell count and neutrophil count were significantly elevated in those with sepsis (*p* < 0.001). Conversely, there was no significant difference in the absolute monocyte count between the two groups (*p* = 0.377). Furthermore, despite the significantly increased full blood count parameters in the sepsis group, no correlation was detected between the cell ratios and procalcitonin. The receiver operating characteristic curve analysis established that the NLR was the best screening marker for sepsis amongst the three cell ratios.

Researchers have demonstrated that an elevated NLR indicates sepsis and predicts morbidity and mortality. For example, a study conducted on 60 000 intensive care patients from the Beth Israel Deaconess Medical Center in the United States between June 2001 and October 2012 concluded that an NLR of > 20.25 in adults was associated with an elevated mortality risk after 28 days.^22^ Although other studies support this finding,^23,24^ controversy over cut-off values remains. In a similar study to ours, a retrospective laboratory analysis of 1468 Turkish patients recommended an NLR cut-off of > 5 as being highly suggestive of sepsis.^35^ In this current study, we defined sepsis based on a positive BC; eight of the 62 (13%) confirmed sepsis patients had an NLR < 5 suggesting that a cut-off value of > 5 is significant. However, an NLR of > 5 cannot be used in isolation to indicate sepsis in Africa due to the high frequency of benign ethnic neutropenia which may result in a lower NLR in healthy individuals.

Although the NLR, in this current study, was significantly increased in patients with sepsis, there was no significant correlation with procalcitonin levels. One reason for this finding could be that the NLR and procalcitonin can be elevated in several conditions, with neither test being specific for sepsis.^17,36^ Neutrophil counts and the NLR can be influenced by numerous variables, including age, obesity, HIV, cardiovascular disease, type 2 diabetes and malignancy.^24^ Both procalcitonin and the NLR measure different processes, and therefore neither should be used in isolation to diagnose or predict the outcome in patients with sepsis.

Although our study revealed no significant difference in the monocyte count between those with and without sepsis, the MLR was significantly elevated in the sepsis group (*p* < 0.001), which was mainly due to a decreased lymphocyte count. This finding implies that the MLR could be an additional valuable indicator of sepsis. Reports examining the use of the MLR are scant and unclear, with one study of 152 patients diagnosed with *Klebsiella pheumonia* in China, between January 2014 and December 2017, demonstrating an association with negative BC^28^ and another prospective analysis of 392 patients diagnosed over a 4-year period in Serbia reporting only a moderate association with sepsis.^29^ Due to these conflicting observations, further studies in different population groups are recommended.

In this current study, as expected, platelet counts in those with sepsis were significantly reduced (*p* < 0.001), while the PLR, although showing a wide variation, was significantly different to the control group (*p* < 0.001). However, receiver operating characteristic curve analysis demonstrated that the PLR only had moderate diagnostic value. These results support two previous research reports, one conducted between January 2011 and July 2013 in the Netherlands and another undertaken between 2001 and 2008 in China, which demonstrated that low platelet count and elevated PLR are associated with sepsis and a worse prognosis in cases of severe thrombocytopenia.^32,33^ Calculating the PLR could provide valuable information if analysed with other cell ratios and full blood count parameters.

### Limitations

In this study, we hypothesised that simple full blood count parameters, utilised together with calculating haematological cell ratios, could provide an early screening tool for sepsis. However, as this was a laboratory-based study, a significant limitation of this work was the lack of clinical information, and therefore we could not link these parameters with disease severity, the sequential organ failure assessment score or outcome. Furthermore, emerging parameters such as the monocyte distribution width were not investigated.

### Conclusion

An automated full blood count and differential are widely available. It is a simple test that is easy to perform, inexpensive and is the first investigation requested by clinicians in the emergency ward. Although procalcitonin and BC remain the gold standard for sepsis diagnosis, they are often only available in central laboratories. Our results suggest that although each full blood count parameter should not be used alone, used together they could provide a simple screening tool for the detection of early sepsis in limited-resource settings.
